# Triboelectric Nanogenerators for Harvesting Diverse Water Kinetic Energy

**DOI:** 10.3390/mi13081219

**Published:** 2022-07-29

**Authors:** Xiaojing Cui, Cecilia Yu, Zhaosu Wang, Dong Wan, Hulin Zhang

**Affiliations:** 1 College of Physics and Information Engineering, Shanxi Normal University, Taiyuan 030031, China; 20210084@sxnu.edu.cn; 2 School of Civil and Environmental Engineering, Georgia Institute of Technology, Atlanta, GA 30332, USA; yu8@gatech.edu; 3 College of Civil Engineering, Taiyuan University of Technology, Taiyuan 030024, China; 4 College of Information and Computer, Taiyuan University of Technology, Taiyuan 030024, China; wangzhaosu0058@link.tyut.edu.cn

**Keywords:** triboelectric nanogenerators, water kinetic energy, self-powered sensors, monitoring water environments

## Abstract

The water covering the Earth’s surface not only supports life but also contains a tremendous amount of energy. Water energy is the most important and widely used renewable energy source in the environment, and the ability to extract the mechanical energy of water is of particular interest since moving water is ubiquitous and abundant, from flowing rivers to falling rain drops. In recent years, triboelectric nanogenerators (TENGs) have been promising for applications in harvesting kinetic energy from water due to their merits of low cost, light weight, simple structure, and abundant choice of materials. Furthermore, TENGs can also be utilized as self-powered active sensors for monitoring water environments, which relies on the output signals of the TENGs caused by the movement and composition of water. Here, TENGs targeting the harvest of different water energy sources have been systematically summarized and analyzed. The TENGs for harvesting different forms of water energy are introduced and divided on the basis of their basic working principles and modes, i.e., in the cases of solid–solid and solid–liquid. A detailed review of recent important progress in TENG-based water energy harvesting is presented. At last, based on recent progresses, the existing challenges and future prospects for TENG-based water energy harvesting are also discussed.

## 1. Introduction

With the rapid development of the economy and the increasing improvement of people’s lives, the issues of massive energy consumption and the accompanying environmental pollution are becoming more and more pressing. The study of green renewable energy harvesting from the environment has thus become a hot research topic. Water covers over 70% of the Earth’s surface, and it not only supports diverse life but also possesses the potential to produce tremendous energy. Water energy involves energy in a variety of forms, including thermal, chemical, nuclear, and mechanical energy. Specifically, the mechanical energy of water is ubiquitous in our environment, since it can be conveyed by water motion or flux in rivers and oceans as well as be generated from rain droplets. Harnessing kinetic energy of water is a promising energy technology in large-scale engineering applications due to both the abundant reserves and little dependence on environmental conditions.

At present, the major technological scheme for extracting hydro-energy and ocean energy is to use an electromagnetic generator (EMG) with various mechanical structures, which is based on Faraday’s law of electromagnetic induction [1,2,3,4]. Although it has been developed for decades, the EMG is still facing great engineering difficulties, including poor scalability and high cost, resulting in electrification in a low exploiting level [1,2,3]. It is necessary to obtain stable and high operating frequency (50−60 Hz) for EMG. However, the frequency of the wave, tide, and current is relatively low (0.1−5 Hz) [5,6]. Meanwhile, various alternative energy scavenging technologies have been developed, such as approaches by photovoltaic [5,6], piezoelectric [7,8,9,10], and thermoelectric [11,12] effects. Especially, the electromagnetic generators usually are huge and immovable, which has several disadvantages, including high cost, huge size and weight, and low mobility. Meanwhile, the piezoelectric generators are working based on piezoelectric polariton induced by mechanical deformation. The common piezoelectric materials are brittle, synthesis-complicated, and even toxic, which greatly limits their applications in a wide range. Among them, the TENG shows the benefits of low cost, light weight, simple structure, and abundant choice of materials [13,14,15,16,17]. The working mechanism of TENGs is based on the conjunction of contact and electrostatic induction. The TENG can effectively extract energy in the frequency range of <5 Hz due to its distinct mechanism [18,19,20,21,22], which makes up for the shortcomings of EMG in obtaining low-frequency mechanical energy and provides a new way to effectively collect ocean energy. The TENG can be dominated by the displacement current derived from the Maxwell equation [23,24]. On the hand, the material wear is fatal for TENGs’ long-term operation, especially for a solid–solid interface. To address this issue, plenty of attention was paid to the fabrication of diverse materials and configurations. In addition, the interfacial charge transfer process is not totally clear, resulting in the relatively lower energy conversion efficiency. Nevertheless, more and more researchers all over the world have studied water energy collection based on TENGs, making the TENG suitable for kinetic energy harvesting from water due to its structural flexibility and adjustable configuration [25,26,27,28,29].

Several reviews have been published on TENG-based water energy harvesting. However, they only focus either on the energy grabbing in a certain application scenario [30,31,32], and others are recapitulative summaries of TENG’s progress in principle [33,34]. Unlike these previous works, in this survey article, TENGs targeting the harvest of different forms of kinetic energy of water have been more systematically summarized and analyzed. This article firstly reviews the structure and properties of the TENGs for harvesting different water energy on the basis of their basic working principles and modes, i.e., in the cases of solid–solid and solid–liquid. Therefore, this review can systematically analyze the application characteristics of solid–solid and solid–liquid TENGs in harvesting water energy. In the next section, an in-depth introduction is provided on the fundamental comprehension of the TENG in water energy harvesting, which is classified as induced by ocean waves, waterdrops and fluids. Subsequently, a detailed review of recent important progress in TENGs for harvesting various water energy is presented. Finally, the challenges and future research trends in the collection of water energy are summarized at the end of the review.

## 2. The Working Modes of TENGs

The TENG device was first invented by Wang’ group in 2012, which aimed to effectively convert mechanical energy into electricity by using Maxwell’s displacement current as the driving force [35,36,37]. The innovative TENGs could not only be used as power sources to drive small electronics but could also serve as active sensors for monitoring the environment. Compared with traditional methods of generating electricity, TENGs can scavenge any types of randomly distributed, irregular, typically wasted low-frequency mechanical energies in the environment based on the coupling of triboelectrification and electrostatic induction, including energy from motions such as vibration [38,39,40], rotation [41,42,43,44], human movement [45,46,47,48], and so on. More than that, by adjusting the configurations, TENGs are well suited for harvesting the kinetic energy induced by water waves [49,50,51], flowing water [52,53,54], and water droplets [55,56,57,58].

In the case of TENGs, triboelectric charges are produced on interfaces due to triboelectrification/contact electrification between two different materials [59,60,61]. Contact electrification occurs for all phases, including solid, liquid, and gas, and it is the most fundamental phenomenon at an interface and plays a fundamental role in physics, chemistry, and biology. So far, the TENGs for harvesting water energy have been demonstrated based on the contact electrification between solid–solid and solid–liquid interfaces.

### 2.1. Fundamental Triboelectrification/Contact Electrification

**Figure 1:**
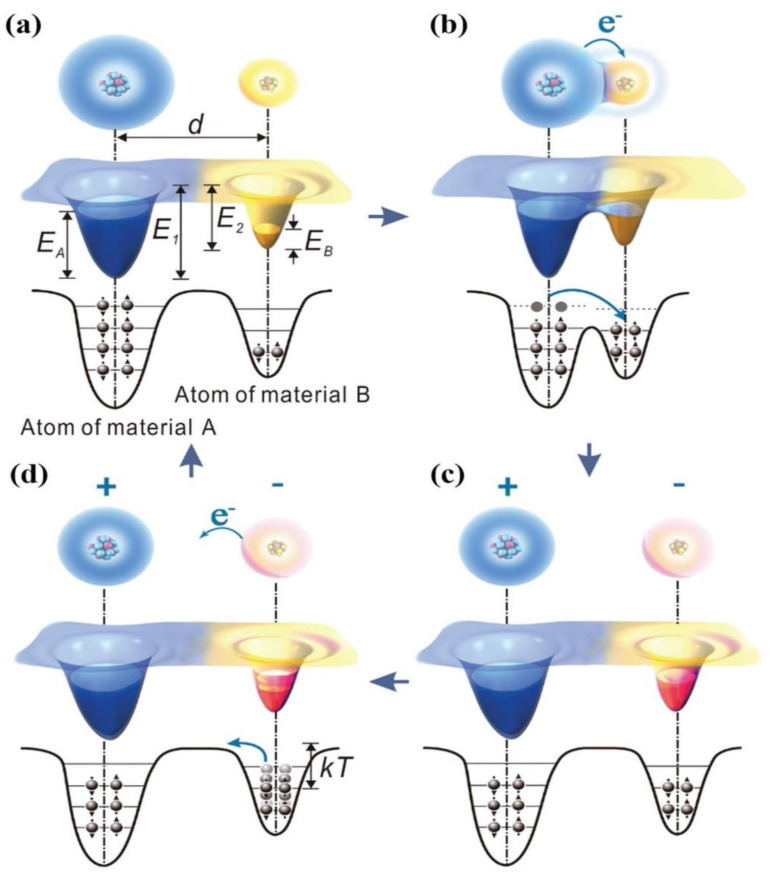
An electron cloud model for contact electrification between two materials (**a**) before contact, (**b**) in contact, (**c**) after contact, and (**d**) the charge release from the atom (reproduced with permission from [66] Copyright 2018 WILEY-VCH Verlag GmbH & Co. KGaA, Weinheim, Germany).

Triboelectrification/contact electrification is an ancient physical phenomenon that has been discovered as early as 2600 years ago in ancient Greek civilization. However, the mechanism of triboelectrification is not clear yet, which may be attributed to electron transfer [62], ion transfer [63] or even material species transfer [64,65]. Recently, Wang et al. conducted a nano-scale study of triboelectric process using Kelvin probe microscopy and revealed that the main operating process of triboelectrification among solids, liquids and gases is electron transfer [66,67,68,69,70]. The electron cloud/potential model on fundamental electron cloud interaction proposed by Wang demonstrates all types of triboelectrification effect [66]. As shown in Figure 1, the electron clouds of the two materials remain separated without overlap before material A and material B come into contact, where the potential well binds the electrons to prevent them from escaping. When the atoms of two materials come close and contact with each other, the electron clouds overlap to form ionic or covalent bonds due to the physical contact. If an external compressive force is applied, the length of the bond will be further shortened, resulting in the initial single potential changing to an asymmetric double-well potential. As the strong electron clouds overlap, the energy barrier between the two atoms is lowered, and electrons can be transferred from one to the other, leading to triboelectrification. In general, when the two materials are separated, the transferred electrons will be kept on the surface of the materials. Once when the temperature increases, the electron energy fluctuations become higher, and the electron transfer will occur across the energy barrier. Overall, TENGs operate by the coupling of triboelectrification and electrostatic induction. When the two different dielectric materials are contacted and separated from each other, due to the different electron affinity, the triboelectric charges will be produced and transferred between each other, resulting in a potential difference. Hence, the alternating electricity can be delivered in the external circuit.

### 2.2. Solid–Solid TENG

**Figure 2:**
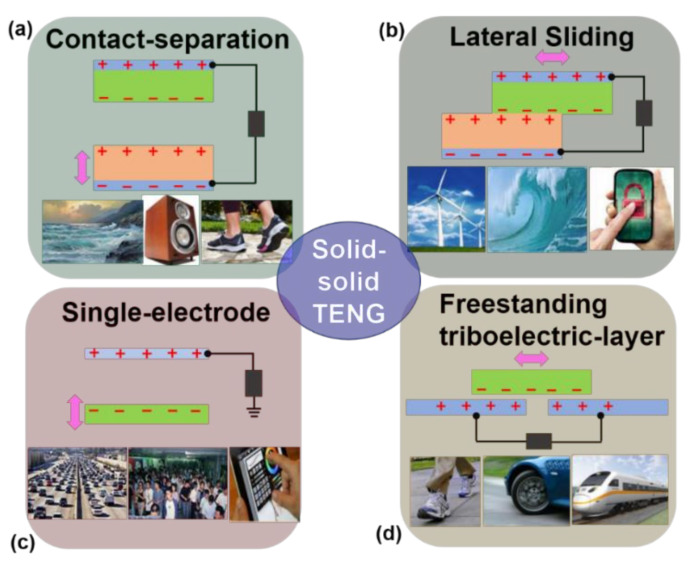
Four basic fundamental modes of solid–solid TENGs: (**a**) contact–separation mode; (**b**) lateral-sliding mode; (**c**) single-electrode mode; and (**d**) freestanding triboelectric-layer mode (reproduced with permission from [38] Copyright 2013 American Chemical Society, New York, NY, USA).

The first TENG was invented as a solid–solid type, which has been widely explored in the recent years. The typical operation principle is based on the coupling of triboelectrification and electrostatic induction between two solid tribo-layers with different triboelectric polarities [13,71,72,73]. The produced tribo-charges on a dielectric interface can remain for a relatively long time, thus actually serving as an induction source for electricity generation on TENGs. As the external mechanical stimulation is applied, the interfacial contact status or relative displacement will deliver a sustainable change that will result in a periodic variation of the induced electric potential difference between the triboelectric pair. In order to maintain the electric equilibrium on the whole device and screen the interfacial potential difference, the free electrons in the back electrodes will flow back and forth alternatively between each other. As long as this cycle keeps up, the applied mechanical motions can be transformed into electricity continuously. With respect to different configurations of TENGs consisting of diverse triboelectric pairs/electrodes and/or different moving manners between triboelectric layers, the solid–solid TENGs can realize energy conversion via the combination of contact electrification and electrostatic induction. As sketched in Figure 2, the four basic fundamental modes of solid–solid TENGs are summarized as follows [30,74].

Contact–separation mode: The simplest design of the TENG is the contact–separation mode (Figure 2a). Two dissimilar dielectric films work as a triboelectric pair that face each other in a stacked structure [75,76,77]. Due to the difference in triboelectric polarity, charges can be injected from one film to the other between interfaces when the two films come into direct contact, resulting in the opposite charges being created on two tribo-layers. Once the two layers that are separated vertically from each other are triggered by a mechanical motion, a potential drop is generated across the two back electrodes coated on dielectric films, which can drive an electron flow through the external circuit. Once the two layers are close, the triboelectric-charge-created potential tends to disappear and, simultaneously, the electrons flow back to deliver the original electrical balance [78].

Sliding mode: The configuration of the sliding mode is the same as that for the contact–separation mode (Figure 2b). Originally, a relative sliding motion in the in-plane direction also creates triboelectric charges on the interfaces when two dielectric films fully contact each other [79,80]. As the two tribo-layers slide outward, a lateral polarization is thus produced along the sliding direction. Electrons on the back electrodes will be driven to flow by the induced interfacial potential in order to balance the electric field introduced by the tribo-charges. A periodic sliding outward and inward produces an AC output. This is the basic sliding mode TENG. The sliding can be achieved from many scenarios, not only planar motions, but also cylindrical rotations [81], or disc rotations [82,83]. This sliding mode can deliver a more effective charge generation between two tribo-layers than the pure contact mode. Furthermore, via a grated design [84,85], the in-plane charge separation in the sliding mode can be enabled several times compared to that in the vertical contact–separation mode. The power output can also be significantly elevated on account of more efficient charge transfer. 

Single-electrode mode: The two modes introduced above have two back electrodes coated on the moving tribo-layers. In a real environment, moving dielectric materials are usually charged because of their contact with air or other surroundings. In these cases, the moving object that is part of the TENG is very inconvenient to be electrically connected to the load, such as a human walking or running on a floor. Therefore, we introduce a single-electrode TENG [86,87], in which only one electrode is attached on one tribo-layer with the other one grounded (Figure 2c). As long as the size of the TENG is finite, a departing or approaching of the bottom object from the single electrode would change the local electrical field distribution, achieving electron exchanges between the electrode and ground to maintain the holistic potential balance. In this mode, the relative movement between the tribo-layers or the tribo-layer and electrode can be obtained from multiple types, including the vertical contact–separation mode [88,89,90], sliding mode [16,91], and even the hybrid mode [92]. This single-electrode TENG is particularly suitable for harvesting energy from moving charged objects or for acting as a self-powered sensor to detecting moving objects. 

Freestanding triboelectric-layer mode: To harvest kinetic energy from a free-moving object charged by the triboelectric effect, the TENG composed of two stationary electrodes is introduced here. Due to the symmetric configuration, a freestanding triboelectric layer alternatively becomes close to either one of the two electrodes so that the induced polarization potential difference between two electrodes will be periodically switched. The required condition that is easy to satisfy is that the size of the electrodes and the gap between them are of similar order with that of the moving freestanding triboelectric layer. Thus, electrons flow between the two electrodes to balance the local potential distribution (Figure 2d) [93,94,95]. The AC output is produced by the oscillation of electrons between the electrode pair. In this mode, the moving layer does not have to contact physically the top dielectric layer of electrodes so that free rotation without direct mechanical contact is possible and wear of the tribo-interfaces can be drastically reduced. This is a good way to improve the TENGs’ durability and reliability. Based on this freestanding layer structure, the energy harvesting from human walking/running and moving automobiles has been extensively demonstrated [96,97,98], showing a great potential application in grabbing energy from free-moving objects without any electric connections.

### 2.3. Solid–Liquid TENG

Based on the water–solid contact electrification, Lin et al. reported a water-based TENG, which is the first solid–liquid TENG [99]. Based on contact electrification and electrostatic induction, the solid–liquid TENG can effectively collect diverse energy from water. It is worth noting that contact electrification between the liquid–solid interface is in the form of the electric double layer (EDL). The classical model for the EDL is the formation of an ion layer on the solid surface, which tends to attract ions with opposite signs while repelling the ions with the same sign in the liquid, establishing an electric potential distribution between the solid and liquid interfaces [33,34,100]. Recently, Prof. Wang at Georgia Tech creatively proposed a two-step formation of the EDL, which is called the Wang model for EDL [70,101,102,103]. The first step is an electron exchange occurring between the liquid and solid surfaces as proposed for contact electrification, which makes the atoms on the solid surface ionized. The second is an ion interaction in the liquid, leading to a gradient distribution of cations and anions near the interface. In practice, experiments proved that the electron exchange and ion adsorption proceed simultaneously and coexist at the liquid–solid interface [104]. Here, the solid–liquid TENGs are simply classified into three modes based on their function and structures.

**Figure 3:**
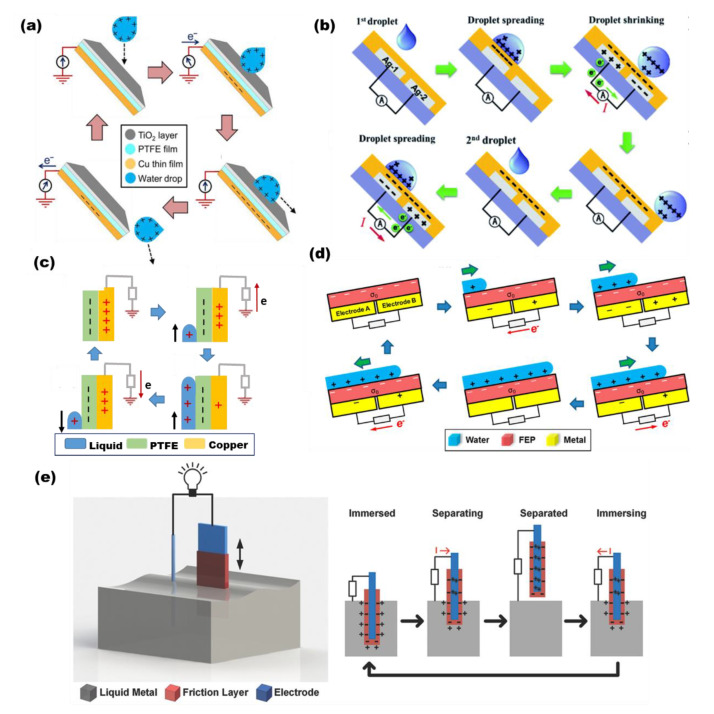
(**a**) Single-electrode waterdrop mode TENG (reproduced with permission from [105] Copyright 2014 WILEY-VCH, Weinheim, Germany). (**b**) Freestanding triboelectric-layer waterdrop mode TENG (reproduced with permission from [107] Copyright 2019 Royal Society of Chemistry, London, UK). (**c**) Single-electrode liquid-column mode TENG (reproduced with permission from [113] Copyright 2019 WILEY-VCH, Weinheim, Germany. (**d**) Freestanding triboelectric-layer liquid-column mode TENG (reproduced with permission from [49]. Copyright 2018 American Chemical Society, New York, NY, USA) (**e**) Liquid–metal–electrode mode TENG (reproduced with permission from [119]. Copyright 2015 WILEY-VCH, Weinheim, Germany).

Waterdrop mode TENG: To harvest waterdrop energy, a solid–liquid TENG based on the process of contact electrification with air/pipes and/or a polymer film was invented by Wang’s group in 2014 [105]. As the waterdrop charged positively near the polytetrafuoroethylene (PTFE) film, there is a positive potential difference generated between the electrode and ground, which can cause the electron transfer and thus deliver a positive current signal (Figure 3a). When a TENG operates through the waterdrop’s contact with the polymer film, the ionization of PTFE surface groups can keep the PTFE negatively charged once the water drops onto the PTFE surface [106,107,108,109]. Meanwhile, the water droplet, owing to its possession of a certain volume, can form a positive EDL and neutralize the induced charges on the contact interface. When waterdrops recede from the PTFE film, the potential difference between the copper electrode and ground will generate a reversed current. These triboelectric charges can be maintained for a long time on the PTFE film because of the intrinsic property of insulators. As long as the water droplets fall continuously, the potential difference can be re-constructed, and electrons will move back to achieve a new electric balance. Therefore, an uninterrupted electric output can be obtained from continuous water droplets. Another waterdrop mode TENG (Figure 3b), a bi-electrode freestanding mode TENG (BF-TENG) is designed by Zhao, which can harvest hydrodynamic energy from rain droplets with regard to the implementation at rooftops, terraces and greenhouse roofs [107].

Liquid-column mode TENG: When harvesting energy from flowing water, the liquid-column mode TENG is a typical choice. The basic electricity generation principle can be explained as a result of contact electrification and electrostatic induction owing to the emerging and submerging of the TENG in liquid, which drives the electron transfer between the triboelectrode and ground [110,111,112]. As illustrated in Figure 3c [113], PTFE, as a highly negative material in the triboelectric series, will possess negative charges at its surface due to contact electrification when the PTFE contacts with liquid (water) [49,114,115,116,117,118]. Conversely, to keep the electrostatic balance, positive charges will be induced on the electrode. When the TENG is gradually inserted into or pulled out of the water, the negative charge layer on the PTFE surface will be partially compensated by forming an EDL, which will induce electrons to flow from the ground to the electrode or from the electrode to the ground, alternatively, to neutralize the unscreened interfacial charges. Another configuration based on an advanced grillage design was reported by Zhu, which is capable of harvesting the fluctuation energy of water waves. When the water moves up and down, the electric dipole moment will screen negative charges on the polymer surface and induce electrons to move back and forth between electrodes (Figure 3d [49]).

Liquid–metal–electrode mode TENG: To improve the total interfacial charge density that can be transferred and thus the total energy conversion efficiency, the liquid–metal–electrode TENG was constructed. As depicted in Figure 3e [119], due to their different triboelectric polarities, the electrons will be injected from the liquid metal to the polymer. As the device moves out of the liquid, the tribo-charges in the interface are separated, which will induce a potential difference between the liquid metal and induction electrode and thus drive electrons to flow to the liquid metal. Similarly, as the TENG slice moves back into the liquid metal, the reversed interfacial potential difference will provoke electrons back. Thus, the entire process will produce an AC electric output. This liquid–metal–electrode mode TENG is well suited for improving output performance [120].

### 2.4. Material Selection of Water-Based TENGs

**Figure 4:**
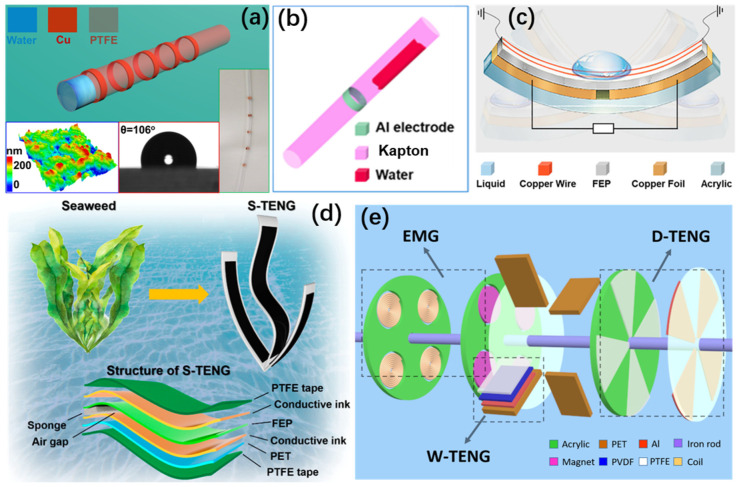
(**a**) Schematic diagram of the tube-based TENG (reproduced with permission from [124]. Copyright 2018 Elsevier Ltd., Singapore). (**b**) Schematic diagram of the TENG (reproduced with permission from [125]. Copyright 2018 Wiley-VCH Verlag GmbH & Co. KGaA, Weinheim, Germany). (**c**) Schematic diagram of the DB-TENG (reproduced with permission from [126]. Copyright 2021 American Chemical Society, New York, NY, USA). (**d**) Schematic diagram of the S-TENG (reproduced with permission from [127]. Copyright 2021 American Chemical Society, New York, NY, USA). (**e**) Schematic diagram of the rotating hybrid nanogenerator (reproduced with permission from [128]. Copyright 2017 WILEY-VCH Verlag GmbH & Co. KGaA, Weinheim, Germany).

The material selection can greatly affect the output performance of TENGs due to the different triboelectric polarities. The common rigid triboelectric materials in TENGs are mainly the metal layer, playing a dual role of the positive triboelectric layer and single electrode. Based on the triboelectric sequence, the usually used positive triboelectric materials include Al, Cu, Ag, Au and carbon, which all have sufficient conductivity and triboelectric performance [121,122,123]. With regard to negative triboelectric materials, diverse polymer films have attracted much attention. Zhang and co-workers studied the characteristics of different high polymer films that were used as negative triboelectric materials, which include fluorinated ethylene propylene (FEP), Kapton and PTFE [124,125,126], as shown in Figure 4a–c. To collect water energy more effectively based on flowing motions, the triboelectric layers of TENGs are always soft or flexible. Under the flowing water or water droplets, the periodic vibration of the flexible textile electrode will convert the water energy to triboelectricity. Wang et al. designed a flexible tube made by Al-coated polyester fabric, as depicted in Figure 4d–e [127,128]. 

In short, the common flexible triboelectric materials are mainly commercial polymer films, which include FEP, Kapton, PTFE, polyethylene terephthalate (PET), polyvinylidene fluoride (PVDF) and so on. Meanwhile, PVDF has multiple functions, which is capable of harvesting water kinetic energy not only by triboelectric effect but also piezoelectric effect [123,124,125,126,127,128]. 

## 3. TENGs for Harvesting Divers Water Energy

Water exists widely in our surroundings, and it contains abundant energy which can be converted to electric power. Due to its many remarkable advantages, including high conversion efficiency, low cost, and light weight [30,119], TENG has been demonstrated to possess huge potential toward harvesting kinetic energy from ocean waves, droplets and water flows [18,49,129,130,131].

### 3.1. TENGs for Harvesting Ocean Waves Energy

The reported TENGs for ocean wave energy harvesting, fabricated in diverse structures and materials, can be categorized into two cases: direct contact between the tribo-surface and water (solid–liquid case) and an encapsulated device relying on solid–solid contact (solid–solid case) [18,72]. The performance of the TENGs is variable due to the structure and friction of the materials adopted.

#### 3.1.1. Solid–Solid TENGs for Harvesting Water Wave Energy

**Figure 5:**
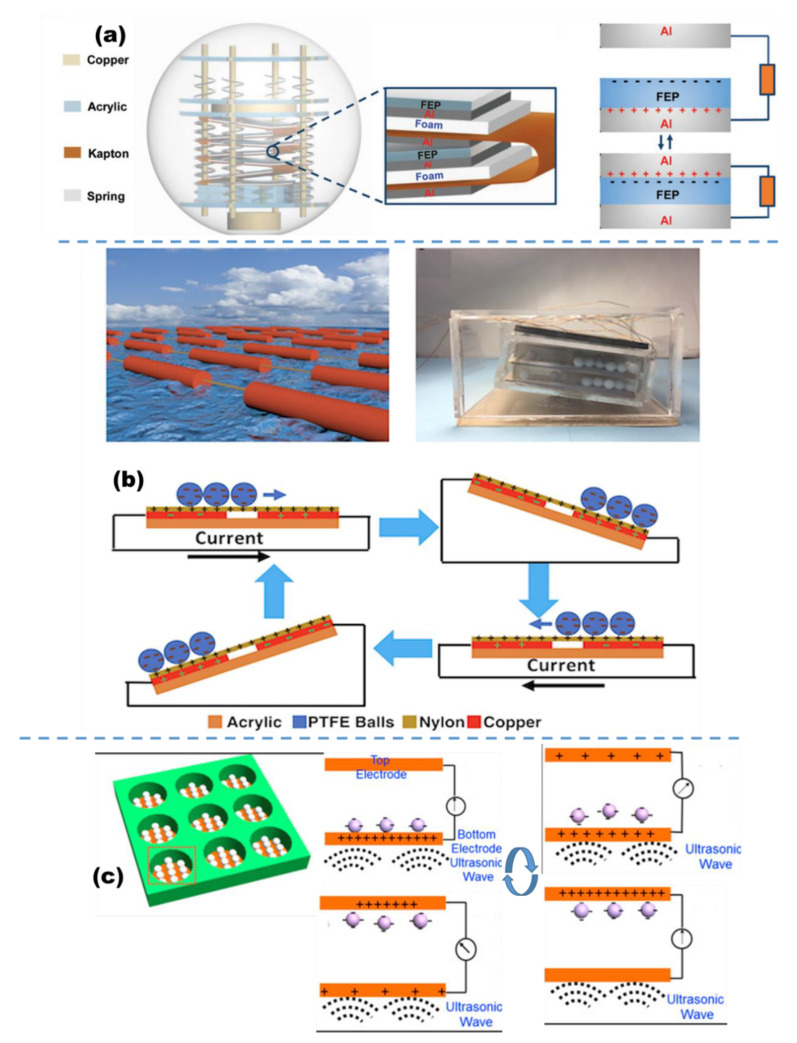
(**a**) A hexagonal TENG network consisting of spherical TENGs units for harvesting water wave energy (reproduced with permission from [131]. Copyright 2019 Elsevier Ltd., Singapore). (**b**) The sea snake-based triboelectric nanogenerator targeted for harvest energy from the wave’s curvature (reproduced with permission from [132]. Copyright 2018 Elsevier Ltd., Singapore). (**c**) TENG targeted for the harvesting of underwater ultrasonic vibrations (reproduced with permission from [133]. Copyright 2017 Elsevier Ltd., Singapore).

Since the first fully enclosed TENGs based on solid–solid contact electrification that could be used to harvest water wave energy were proposed by Wang et al. [117], more and more solid–solid TENGs were fabricated to harvest wave energy [49,119,120]. Liang et al. reported a hexagonal TENG network consisting of spherical TENGs units for harvesting water wave energy [131]. As displayed in Figure 5a, the basic TENG units are fabricated based on a spring-assisted multilayered structure, which is based on the contact–separation between the aluminum (Al) electrode and polarized FEP film bonded by another aluminum electrode. Under the triggering of water waves, the contact between the Al electrode and FEP film generates opposite charges on the Al and FEP surface. Their separation then produces an electric potential difference between the electrodes, driving the free electron flowing at the external circuit. The periodic contact–separation will generate alternating currents. With the help of the springs and the integration of multiple basic units, the output of the TENG network can attain the highest outputs of 270 μA and 354 V. When the TENG network is integrated with a power management module (PMM), a steady and continuous DC voltage will be produced on the load resistor. In addition, a digital thermometer and a wireless transmitter were successfully powered by the power-managed TENG network, showing the potential application of TENGs for harvesting ocean energy and remote sensing.

In another work, a sea snake-based triboelectric nanogenerator (SS-TENG) structure is presented by Zhang [132]. The SS-TENG is the first TENG to harvest energy from the wave’s curvature and depends on the advantage of a connected section, which can flex and bend easily as a wave passes through. The SS-TENG is based on a freestanding rolling mode, and the interior is comprised of PTFE balls, nylon film, a sputtered copper layer, and a soft tampered spring (Figure 5b). Under a water wave moving across the acrylic structure, the springs attaching the acrylic boxes would bend, and the acrylic boxes would be inclined due to the curvature of the incoming water wave, causing the balls to roll along the nylon film. After the first contact, the PTFE balls would become negatively charged due to contact electrification with the thin film. As the PTFE balls act as a dielectric layer above the electrodes, a charge transfer will occur between the electrodes due to the balls moving. The TENG has a maximum power density of 3 W/m^3^ under linear motor actuation to simulate ocean waves. The SS-TENG could be easily fabricated, making it a cost-effective and sustainable ocean wave energy harvester.

Not only can the TENG be used to harvest wave energy at the ocean surface, the TENG also can harvest ultrasonic waves underwater. Xi et al. designed a high output current, high-efficiency and low-cost TENG for harvesting energy from underwater ultrasonic waves [133]. The TENG works based on a freestanding triboelectric-layer mode composed of PTFE pellets and two parallel Cu electrode plates fixed on an insulated cubic acrylic plate with the cylindrical hole (Figure 5c). The PTFE pellets directly collide up and down between the two electrode plates when the TENG is placed in an underwater environment with ultrasonic waves. Therefore, the TENG can achieve instantaneous electricity output when the PTFE pellets contact/separate with the two electrode plates under ultrasonic stimulation. The design parameter corresponding to better outputs is 9 holes filled with 7 pellets with a diameter of 3.3 mm. Under an input ultrasonic wave of 80 kHz and 1.38 W/cm^2^, the output voltage and current can reach 170 V and 0.12 A, respectively, with the largest output power of 0.362 W/cm^2^, having the power conversion efficiency of 13.1%. The output equivalent galvanostatic current can achieve 1.43 mA, which is the largest one reported thus far.

#### 3.1.2. Solid–Liquid Teng for Harvesting Water Wave Energy

**Figure 6:**
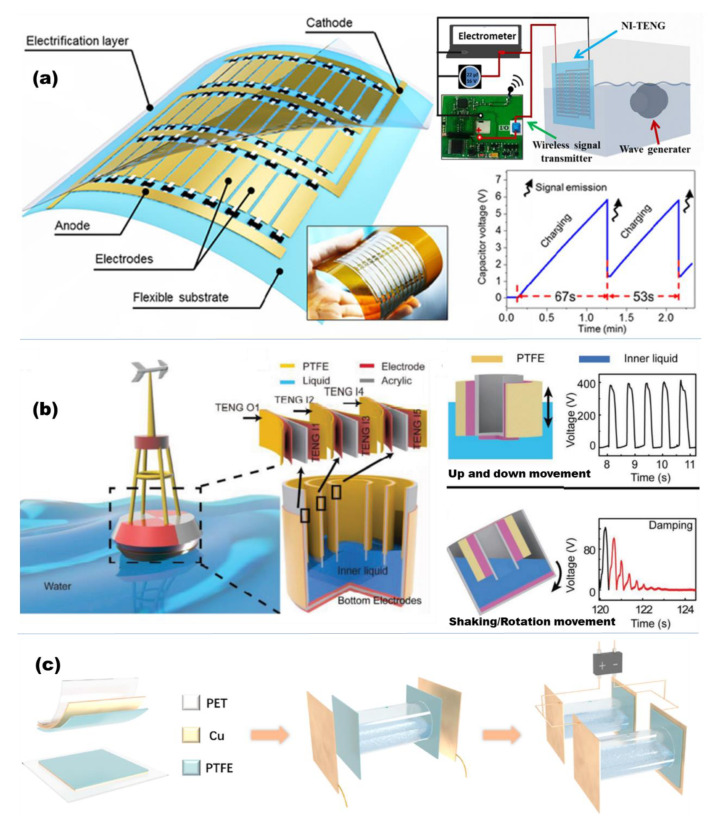
(**a**) The networked-integrated L-TENG and working principle (reproduced with permission from [117]. Copyright 2018 American Chemical Society, New York, NY, USA). (**b**) The buoy-like TENG and electrical characterization (reproduced with permission from [118]. Copyright 2018 WILEY-VCH Verlag GmbH & Co. KGaA, Weinheim, Germany). (**c**) The fabrication procedure and working mechanism of the solid-liquid TENG (reproduced with permission from [130]. Copyright 2020 Elsevier Ltd., Singapore).

The first liquid–solid TENG for harvesting water wave energy was reported in 2013 [99], in which the water contact was explored as one type of “material” choice for TENGs used to harvest water wave energy. So far, a group of TENG devices based on liquid–solid contact electrification has been developed [117,118]. Xue et al. fabricated a networked integrated triboelectric nanogenerator (NI-TENG) for harvesting energy from interfacing interactions with various types of water waves [117]. In this device, multiple pairs of electrodes were fabricated on top of a flexible substrate (Figure 6a). A conductive textile was employed as the electrode material, and a dry-etched PTFE film was used for the hydrophobic coating. Due to a two-dimensional networked structure, the NI-TENG can accommodate diverse water wave motions and generates a stable electric output regardless of the wave type. This merit promises practical applications of the NI-TENG in real circumstances, where water waves are highly variable and unpredictable. Meanwhile, a NI-TENG having an area of 100 × 70 mm^2^ can produce a stable short-circuit current of 13.5 μA and corresponding electric power of 1.03 mW at a water wave height of 12 cm. In addition, the electricity generated was successfully stored and released to power wireless signal transmission, which proves the practical use of the NI-TENG as a power supply for sensor nodes in a wireless sensing network. This work indicates that TENGs can be potentially implemented for regular marine hydrology monitoring, locating/tracking, pollution detection and so on.

In another work, a buoy-like liquid–solid-contact triboelectric nanogenerator (LS TENG) was fabricated to capture the low-frequency water wave energy [118]. As illustrated in Figure 6b, the LS TENG contains two types TENGs: the outer TENG O1 (with the bottom electrode outside) and the inner TENGs (I1 to I5 with the bottom electrode inside of the buoy). Under up–down movement, the LS TENG can harvest ambient wave energy into electricity. While under shaking or rotation movement, the inner TENGs are connected in parallel to collect the mechanical energy of the inner liquid (e.g., water). The network of TENGs could convert large quantities of mechanical energy into electricity to supply portable electric devices, navigation systems, or even a town. The output current and voltage of the network of 18 LS TENGs are 290 μA and 300 V.

Sun fabricated a new type of array combination for liquid–solid TENGs by using PTFE ultrafiltration membrane as the friction layer with water to collect wave energy to self-power the cathodic protection system in marine environment [130]. As shown in Figure 6c, the TENG structure is composed of PET tubes, Cu films, PTFE filtration membranes and distilled water. Due to the structure, the dual liquid–solid TENGs array can generate a short current circuit of 2.68 mA and output voltage of 105 V.

A summary table comparing the works applying TENG in wave energy harvesting in terms of structure design, triboelectric material selection, and power output is given in Table 1. It can be seen from the table that the solid–solid mode TENGs are more flexible and diverse in terms of structure and material selection than the solid–liquid mode, and the outputs of solid–solid mode are relatively larger under the same volume. Therefore, well-sealed solid–solid mode TENGs are better suited to harvesting water wave energy than solid–liquid mode TENGs.

### 3.2. TENGs for Harvesting Waterdrops/Raindrops Energy

**Figure 7:**
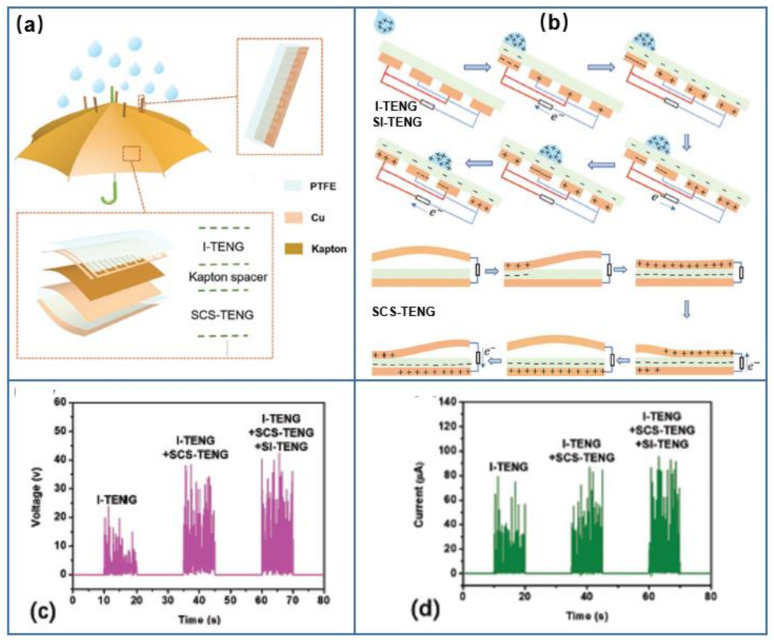
(**a**) The structure, (**b**) working principle, (**c**) output voltage and (**d**) output current of the integrated TENG as one part of an umbrella for rain energy harvesting (reproduced with permission from [146]. Copyright 2019 WILEY-VCH Verlag GmbH & Co. KGaA, Weinheim, Germany).

Rainfall is a very familiar natural phenomenon which is important for the Earth’s water cycle. Moreover, raindrop energy is also a kind of renewable energy source which contains two types energy: the kinetic energy from the gravitational potential energy of falling raindrops and the electrostatic energy generated by contact friction with the air and/or dielectric materials. To harvest raindrop energy, the TENG should always be fabricated with waterdrop mode TENG. The raindrop mode TENGs with different structures and materials have been fabricated [141,142,143,144,145,146,147]. A highly integrated TENG, fabricated as one part of an umbrella (Figure 7a), has been developed for simultaneously harvesting all the forms energy that exist in raindrops [146]. The integrated TENG consists of a solid–solid contact–separation mode TENG (SCS-TENG) for harvesting the kinetic energy from raindrops, a freestanding TENG with interdigitated electrodes (I-TENG) attached to the upper surface of the SCS-TENG, and an array composed of strip-shaped I-TENGs (SI-TENG) standing on the surface of I-TENG used to harvest the electrostatic energy of raindrops. When raindrops fall, part of them will meet SI-TENG, which will harvest the electrostatic energy of these raindrops, and most of the raindrops will knock on the sealed bag packaged by I-TENG and SCS-TENG, which can simultaneously collect kinetic and electrostatic energy from them. In addition, the integrated sealed-bag TENG can also collect the energy of raindrops that slide down from the SI-TENG. Thus, the different kinds of energy carried by raindrops are all scavenged through the integrated TENG. 

The working principle of the integrated TENGs is shown in Figure 7b. Due to the contact electrification with air or drifting particles, raindrops will cause triboelectric charges when they fall from the sky [147]. An electrostatic field can be formed between the PTFE friction layer and charged raindrops. Once the raindrops begin to approach the PTFE with electrodes on its back, under application of the electrostatic field, the external load will generate a forward current until the raindrops are in full contact with the PTFE and the electrostatic field disappears. When the raindrops begin to leave the PTFE, the electrostatic field appears again, and the external load will produce a reverse current. Therefore, the changeable electrostatic field will drive electrons to flow back and forth between the interdigital electrodes through the external load and form an alternating output. In addition, the contact–separation mode TENG adopts a sealed saccular structure with an appropriate amount of gas instead of an intermediate spacer layer. When the falling raindrops impinge on the SCS-TENG, the two friction layers are in contact with each other. As the raindrops slide, the gas in the saccular structure moves, causing a change in the contact area between these two friction layers. Once the two Cu electrodes are electrically connected by a load, the free electrons will flow between the Cu electrode under the PTFE and the upper Cu electrode to balance the electrostatic field built by the triboelectric charges, which will continue until the two friction layers are completely in contact or separated. The performances of the integrated TENG are characterized in Figure 7c,d. The output voltage can reach 42.2 V when the I-TENG, the SCS-TENG and the SI-TENG were connected in series. The highest current of 95.4 μA is obtained when the I-TENG, the SCS-TENG and the SI-TENG were connected in parallel.

**Figure 8:**
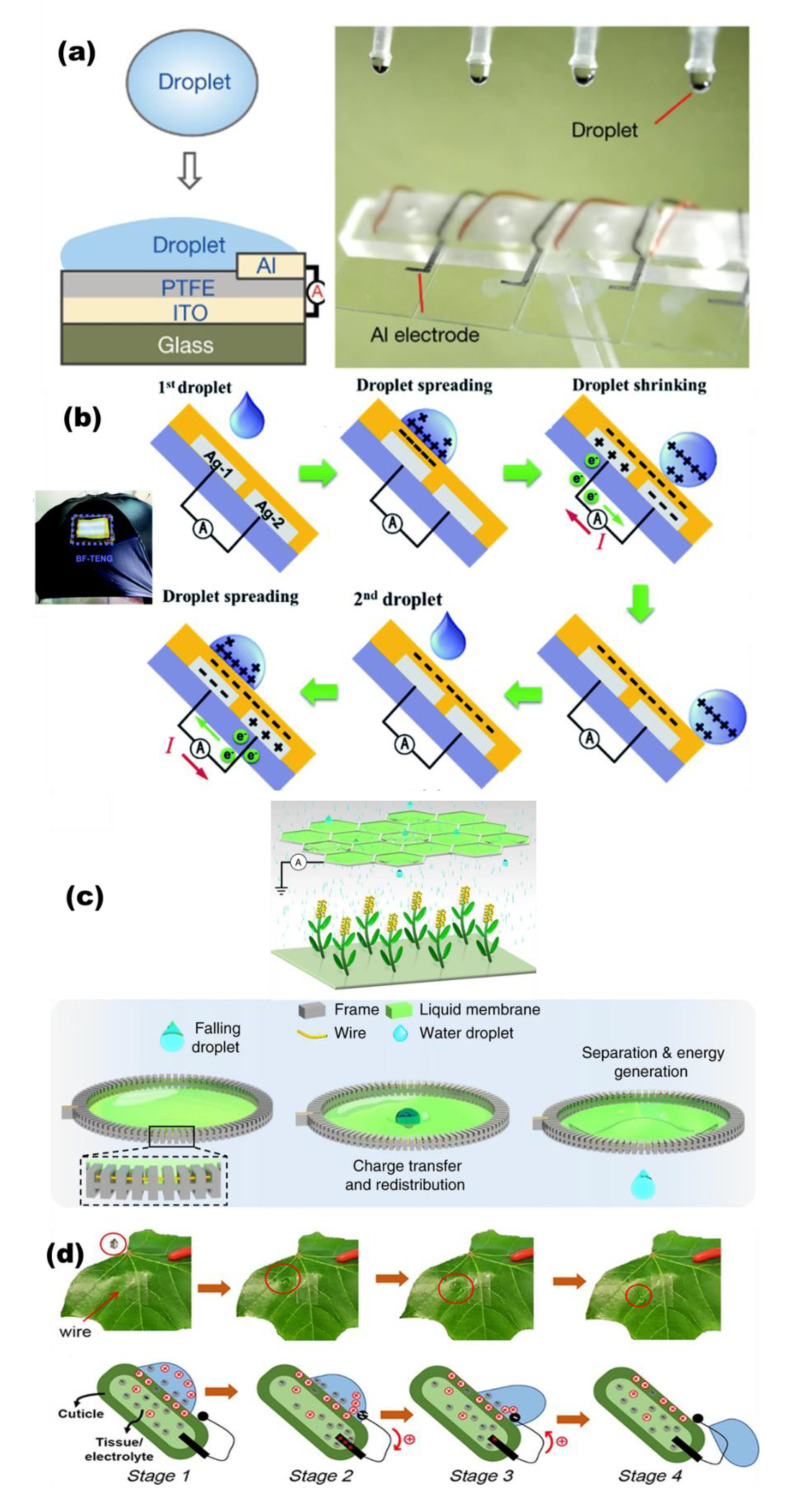
(**a**) The schematic diagram of DEG device (reproduced with permission from [55]. Copyright 2020 Springer Nature, Berlin, Germany). (**b**) The electrification mechanism of water droplets falling on an inclined BF-TENG (reproduced with permission from [107]. Copyright 2020 Royal Society of Chemistry, London, UK). (**c**) The working principle and schematic diagram of L–L TENG collects energy from raindrops in an irrigation system (reproduced with permission from [56]. Copyright 2019 Springer Nature, Berlin, Germany). (**d**) Schematic of the working mechanism of the FBD-TENG (reproduced with permission from [28]. Copyright 2020 American Chemical Society, New York, NY, USA).

In order to improve the efficiency and adapt to different application environments of water droplet TENGs, a variety of TENGs with different structures are fabricated. As shown in Figure 8a, a droplet-based electricity generator (DEG) was developed to harvest energy from impinging water droplets by using an architecture that comprises a PTFE film on an indium tin oxide substrate plus an aluminum electrode [55]. The spreading of an impinged water droplet on the device bridges the originally disconnected components into a closed-loop electrical system, transforming the conventional interfacial effect into a bulk effect, and so enhancing the instantaneous power density by several orders of magnitude over equivalent devices that are limited by interfacial effects. A bi-electrode freestanding mode TENG (BF-TENG) is designed to harvest hydrodynamic energy from rain droplets with regard to the implementation at rooftops, terraces and greenhouse roofs (Figure 8b) [107]. Through coupling of the cumulative charging behavior and the optimal configurations of BF-TENG, an instantaneous maximum power density of 1.838 W m^−2^ is achieved, and 30 LEDs can be lit up when spraying tap water from a shower faucet on a BF-TENG installed on an umbrella. A liquid–liquid triboelectric nanogenerator (L-L TENG) is achieved by passing a liquid droplet through a freely suspended liquid membrane (Figure 8c) [56]. The falling of a droplet (about 40 μL) can generate a peak power of 137.4 nW by passing through a pre-charged membrane. A fully biodegradable TENG (FBD-TENG) whose elements were all made from natural substances was fabricated (Figure 8d) [28]. The leaf cuticle and the inside conductive tissue are utilized as the tribo-material and electrode for one part in the FBD-TENG, and water droplets are employed as the counterpart. 

### 3.3. TENG for Harvesting Water Flow Energy

**Figure 9:**
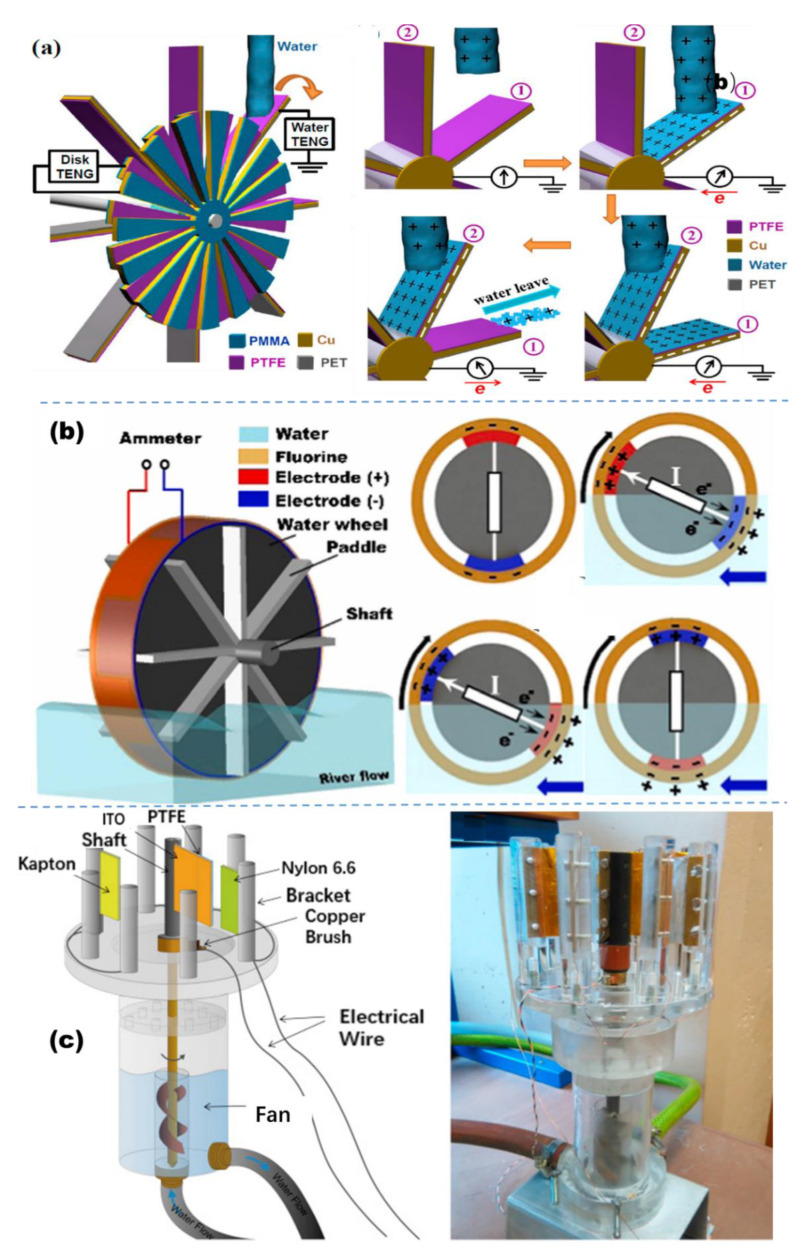
(**a**) The structure of the hybridized TENG (reproduced with permission from [112] Copyright 2014, American Chemical Society, New York, NY, USA). (**b**) Schematic and working principle of the w-TENG (reproduced with permission from [148]. Copyright 2019, Elsevier Ltd., Singapore). (**c**) Schematic diagram of the rotary TENG (reproduced with permission from [54]. Copyright 2016, Elsevier Ltd., Singapore).

Water flow is also an important type of hydropower resource. It typically appears in the forms of rivers, pipe flows, and so on. TENGs are usually designed for harvesting the triboelectric energy from a water stream based on the single-electrode or free-standing triboelectric layer mode [148,149,150]. A water wheel hybridized TENG, composed of a water-TENG part and a disk-TENG part, has been developed for simultaneously harvesting the two types of energies from the tap water flowing from a household faucet (Figure 9a) [104]. The short-circuit current of the water-TENG and the disk-TENG at a flowing water rate of 54 mL/s can reach 12.9 and 3.8 μA, respectively. In another work, another scalable water wheel triboelectric nanogenerator (ww-TENG) was reported for harvesting the low-speed water flow energy (Figure 9b) [148]. The ww-TENG is half immersed in a flowing river and driven by the continuous water flow. The flow motion of the river induces a rotation of the w-TENG; thus, the fluorinated carbon paper surface alternatively contacts and separates with the water, presenting a peak power of 5.3 μW when the load resistance is 50 MΩ. A non-contact cylindrical rotating solid–solid TENG was developed to harvest flow energy from water in pipes [149]. The TENG was composed of two coaxial hollow cylinders. The outer hollow cylinder was the rotor with two pieces of nanowire (NW)-structured FEP thin films symmetrically attached on its inner wall. The inner hollow cylinder (the stator) had four pieces of Cu foils on its outer wall, where the two pairs of Cu foils at the opposite position were connected and act as the two electrodes of the TENG (Cu1 and Cu2). As this cylindrical TENG rotates, driven by a current of water, it could produce continuous electrical output. What is more, the peak open-circuit voltage and short-circuit current of the TENG reached 1670 V and 13.4 μA, respectively under a water flow of 4 L/min. 

Rodrigues fabricated a rotary TENG for harvesting energy from water flows, in which PTFE and Nylon 6.6 are employed as triboelectric materials (Figure 9c) [54]. The operation mechanism of the fabricated rotary TENG is a hybridization of the contact and sliding modes based on the triboelectrification and electrostatic induction effects. The rotary TENG can generate a mean voltage value of ≈102.2 V, a short-circuit current density ≈120 mA/m^2^ and a maximum power density of ≈6.1 W/m^2^. 

To harvest hydrokinetic energy in shallow water with low flow velocity, a three-dimensional (3D) fully-enclosed triboelectric nanogenerator (FE-TENG) with bionic fish-like structure for harvesting hydrokinetic energy is reported [150]. The FE-TENG consists of three parts: the triboelectric power generation unit, bionic fish-like structure and connection unit. Due to the bionic structure, the FE-TENG can realize zero head power generation in shallow water with low flow velocity. The peak power density of the FE-TENG can reach 7 and 0.36 W/m^3^, respectively, under the simulated swing state with a frequency of 1.25 Hz and simulated river current with a flow velocity of 0.81 m/s.

A summary table comparing the works applying TENG in harvesting flowing water in terms of structure design, triboelectric material selection, and power output is given in Table 2. As shown in Table 2, the output voltage of the solid–solid mode TENG is higher than that of the solid–liquid mode TENG, but the output power is relatively small. 

### 3.4. Hybrid Nanogenerators for Harvesting Water Energy 

To harvest water energy more efficiently and enhance the power output, the TENG technology can also be hybridized with other technologies to achieve a hybrid generator [125,128]. As is well known, an electromagnetic generator (EMG) based on the electromagnetic induction also can be utilized to convert water kinetic energy into electrical energy. In addition, piezoelectric nanogenerators (PENG) have been widely studied as another kind of mechanical energy collection technology, which is also considered to harvest water kinetic energy [151,152]. A series of researchers have shown that the integration of EMG/PENG and TENG can be an effective way to improve the device’s energy conversion efficiency.

**Figure 10:**
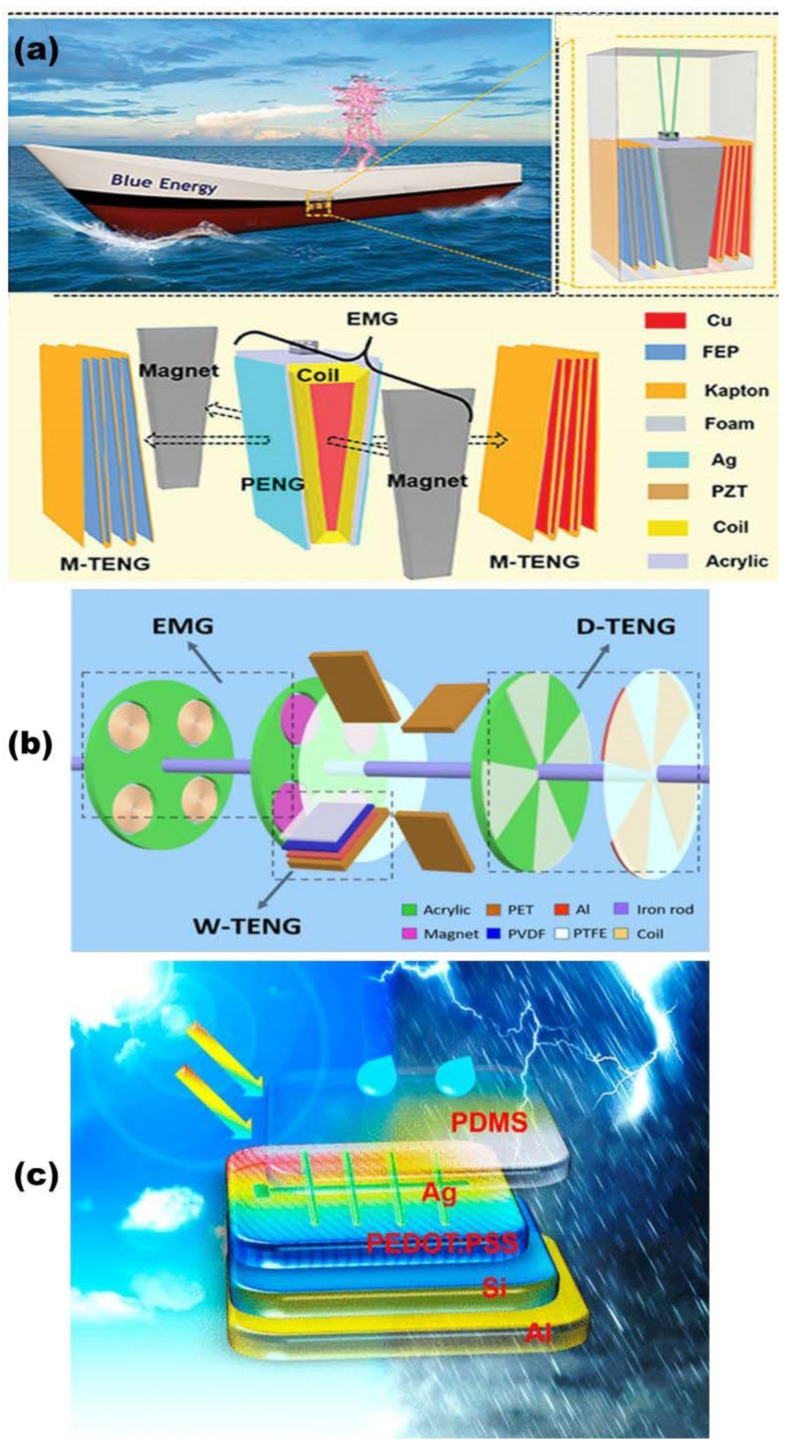
(**a**) Schematic diagram of bifilar-pendulum coupled hybrid nanogenerator (reproduced with permission from [151]. Copyright 2022, Wiley-VCH GmbH, Weinheim, Germany). (**b**) Schematic diagram of the rotating hybridized TENG (reproduced with permission from [128]. Copyright 2017 WILEY-VCH Verlag GmbH & Co. KGaA, Weinheim, Germany). (**c**) The hybrid nanogenerator that integrates a solar cell and a TENG (reproduced with permission from [152]. Copyright 2018, American Chemical Society, New York, NY, USA).

To harvest wave energy, a bifilar-pendulum coupled hybrid nanogenerator (BCHNG) was fabricated, which consists of an EMG, two PENGs, and two M-TENGs, as depicted in Figure 10a [151]. The BCHNG module can harvest the kinetic energy and gravitational potential energy of ocean waves at the same time, which is based on the swing of the bifilar-pendulum. In addition, benefiting from the reasonable utilization of space by coupling PENG and EMG, the BCHNG module obtains an excellent output performance with a power density of 358.5 W m^−3^.

In addition, a rotating hybridized nanogenerator has been established, comprising a water-TENG (W-TENG), a disk-TENG (D-TENG), and an electromagnetic generator (EMG) (as shown in Figure 10b), which has been explored for simultaneously harvesting energies from flowing water and wind [128]. When the flowing water impacted on the wheel blades, the W-TENG begins to work, and the mechanical energy from the water causes the rotation motion of D-TENG and EMG. When the water flow rate is 60 mL s^−1^, the short circuit current and open circuit voltage of the hybrid nanogenerator can reach 7 mA and 10 V, respectively. Experimental results show that the output performance of the composite generator is better than that of TENG and EMG alone.

The integration of the TENG with solar cells is also a good choice. Cao et al. developed a hybrid energy harvesting structure that integrates a solar cell and a TENG device, as shown in Figure 10c, which can generate power from both sunlight and raindrops [152]. The hybrid energy harvesting system integrated electrode configuration can combine the advantages of high current level of a solar cell and high voltage of a TENG device, promising an efficient approach to collect energy from the environment in different weather conditions.

## 4. Characteristics of Water-Based TENGs

### 4.1. Power Management of Water-Based TENGs

**Figure 11:**
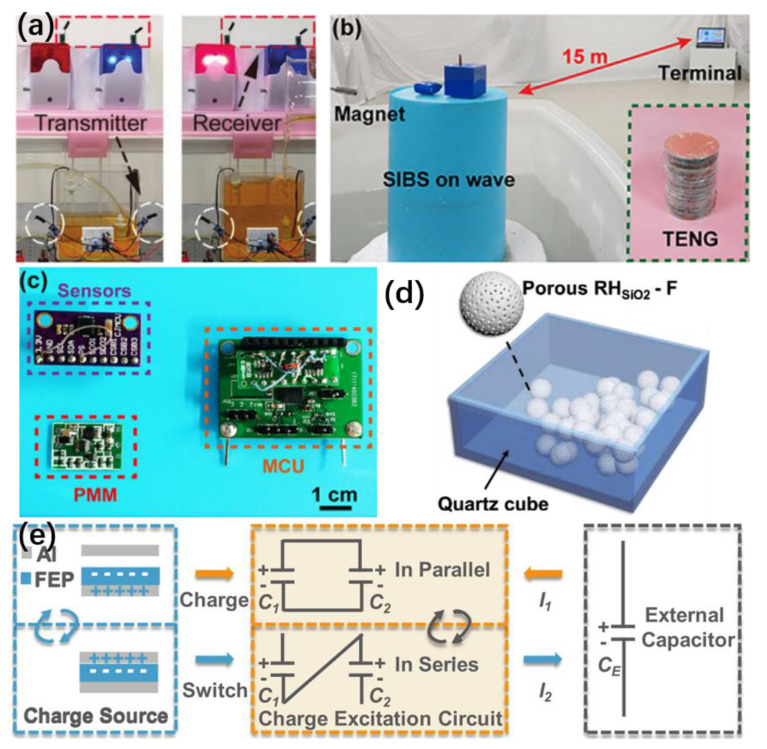
(**a**) Photograph of low and high-water level alarm (reproduced with permission from [153]. Copyright 2021 Wiley-VCH GmbH, Weinheim, Germany). (**b**) Working principle of a contact-separation TENG integrated with the CEC. (**c**) The photos of the PMM, MCU and sensors (reproduced with permission from [154]. 2020 Wiley-VCH GmbH, Weinheim, Germany). (**d**) Schematic diagram showing the RH_SiO2_-F particles encapsulated in a quartz cube (reproduced with permission from [155]. Copyright 2019 Elsevier Ltd., Singapore). (**e**) Demonstration of the SIBS in a simulated wave environment. The inset is the photo of the multilayered TENG (reproduced with permission from [156]. Copyright 2019 Elsevier Ltd., Singapore).

Due to the discontinuity and environmental dependencies of water flows/waves, TENGs for harvesting water energy usually require external electronic components to implement specific applications. For example, by charging capacitors after rectifying the signals generated from the water-based TENGs, power can be stored to drive the signal receiver and transmitter. Chen et al. proposed a TENG with a waterwheel-like structure for water flow energy harvesting and implemented self-powered wireless water level monitoring and warning (Figure 11a) [153]. The height of the water level can influence the switch, which in turn controls the circuit on and off. The transmitter is energized by a 1 mF capacitor with stored electrical energy and sends an acoustic-optical water level alarm signal to the receiver. Capacitors solve the problem that TENGs are inefficient in supplying power directly to electronics because their internal impedance does not match the impedance of most general-purpose electronics. Liang et al. developed a new type of charge excitation circuits (CECs), which are capacitor banks consisting of two identical capacitors C_1_ and C_2_ (10 μF) that can be switched autonomously from parallel to series connection (Figure 11b). [154] During the separation of TENG, the two capacitors are charged in parallel, while during contact, the TENG no longer charges the capacitors but rather switches their connection to series, doubling the voltage of the capacitor bank. The TENG network with CECs for water wave harvesting charges the capacitors (470 µF) and then connects the wireless transmitter to the system. After the RF signal is sent, the corresponding receiving terminal works to control the microcontroller and sends data to the cell phone through the serial port immediately. Xi et al. fabricated a smart buoy system that is powered by water wave energy and can act as a wireless sensor network node to measure acceleration, magnetic strength and temperature. The energy management module (PMM) in this system consists of electronic components integrated on a circuit board, making it more compact and portable (Figure 11c). [154] The energy collected by the TENGs can drive a micro-programmed control unit (MCU), several micro-sensors and a transmitter through the PMM, improving the efficiency of energy transfer. A microcontroller based on an intelligent monitoring mechanism can deploy electrical energy for each sensor according to different priorities and data transmission cycles. It is worth noting that the TENG unit is packaged in a waterproof cylindrical shell with a small size of Φ15 cm × 7 cm (Figure 11d). [155] Durability tests have shown that the multilayer TENG has excellent performance over long periods of operation, with both open-circuit voltage and short-circuit transfer charge holding up well after 24,000 cycles at a frequency of 2 Hz. Due to the volatile and wet nature of the water environment, encapsulation is used to improve the TENGs’ resistance to environmental disturbances and durability. Lai et al. demonstrated a self-powered wireless triboelectric vibration sensor made from the naturally nanoporous SiO_2_ particles which were encapsulated in a quartz cube particle for allowing the detection of the vibrations and movement in the underwater environment (Figure 11e) [156]. In this package condition, the sensor can work stably at various temperatures in the underwater environment. 

### 4.2. Stability, Reliability and Durability of Water-Based TENGs

As a kind of renewable energy, water widely exists in our life, such as in the form of raindrops, rivers and oceans. As a new technology, triboelectric nanogenerators (TENG) can convert energy from water to electricity. Therefore, TENG-based water energy collection has been the focus of research in recent years. However, there are some limitations in corrosion resistance, stability, reliability, and service life in TENG, which have been extensively studied by numerous researchers.

**Figure 12:**
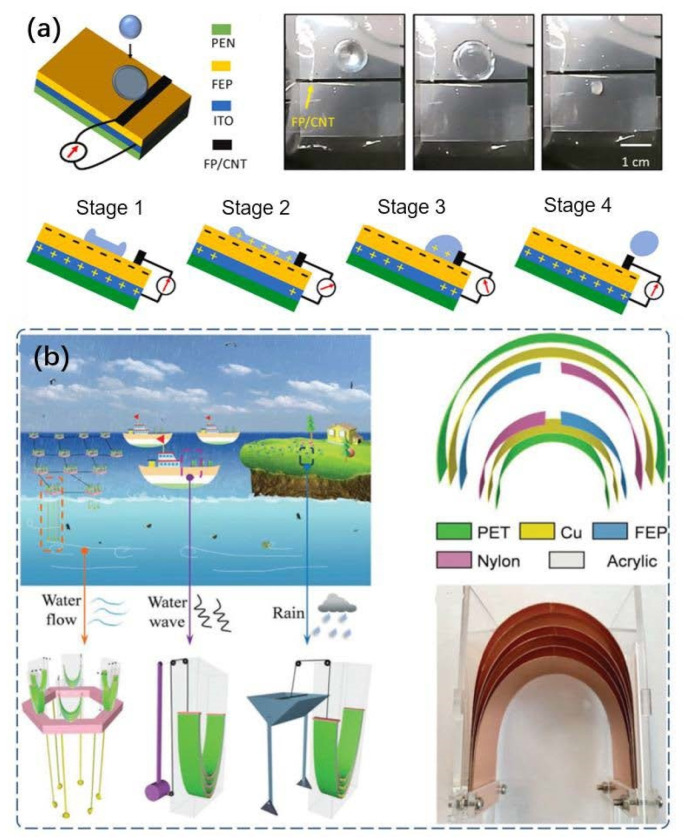
(**a**) A droplet-based electricity generator (DEG) and self-powered electronic skin have been fabricated with a fluoropolymer (FP)/carbon nanotube (CNT) (reproduced with permission from [157]. Copyright 2021 Elsevier Ltd., Singapore). (**b**) A non-encapsulated polymorphous U-shaped triboelectric nanogenerator (NPU-TENG) for multiform hydropower harvesting with high robustness and corrosion resistance (reproduced with permission from [158]. Copyright 2021 Wiley-VCH GmbH, Weinheim, Germany).

Metallic materials can be used as triboelectric materials or conductive electrodes for TENG. However, in the process of collecting water energy, it is very susceptible to corrosion. As depicted in Figure 12a, it reports the development of chemically inert and superhydrophobic electrode based on fluoropolymer (FP)/carbon-nanotube (CNT), which can avoid unwanted corrosion, deformation and damage of metal electrodes in harsh environments [157]. The electrode and the hydrophobic triboelectric material constitute a TENG for collecting droplet energy, which can generate an instantaneous current of 2 mA and an instantaneous power of 0.12 W. Of course, to prevent electrodes from being corroded, it is not only the electrode that needs to be treated, but also the structure of TENG can be optimized. As shown in Figure 12b, a non-encapsulated polymorphous U-shaped TENG for multiform hydropower harvesting with effective robustness is designed, which contains two joinable parts: one U-shaped multilayered TENG unit and three trigger ends chosen according to actual requirements [158]. 

**Figure 13:**
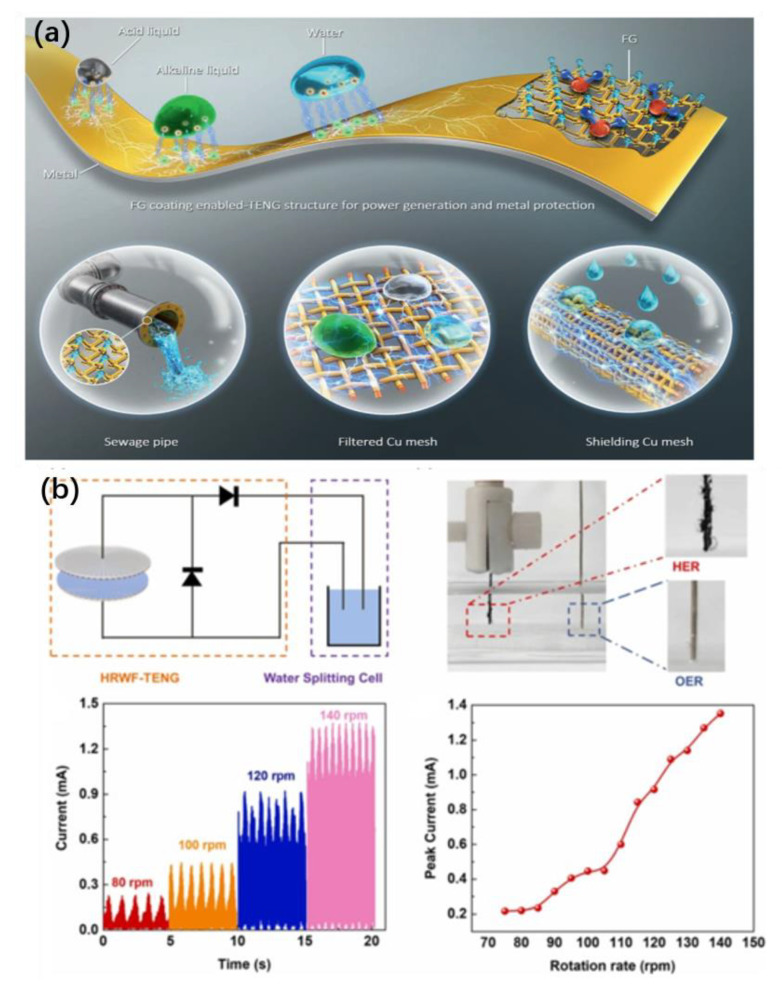
(**a**) Fluorinated graphene-enabled durable triboelectric coating for water energy harvesting (reproduced with permission from [159]. 2021 Wiley-VCH GmbH, Weinheim, Germany). (**b**) A half-wave rectifying triboelectric nanogenerator for self-powered water splitting toward hydrogen production (reproduced with permission from [160]. 2021 Elsevier Ltd., Singapore).

In collecting water energy, the durability and reliability problems of triboelectric nanogenerators still limit their practical applications in long-term operation. In order to improve this situation, fluorinated graphene (FG) with unique triboelectric negativity and superhydrophobic property is introduced to serve as a new triboelectric and protective layer for TENG construction in Figure 13a [159]. It also significantly improves the durability and reliability of TENG for metal protection and water energy collection under extreme conditions such as strong acid and alkali environments. There is an endless supply of water energy, but how to actually use it to produce sustainable energy output is a challenge. As depicted in Figure 13b, researchers reported a half-wave rectifying water flow-driven triboelectric nanogenerator (HRWF-TENG), which can achieve high voltage and low loss DC output, making the open circuit voltage and short circuit current reach 100 V and 90 μA at 140 RPM, respectively [160]. By connecting HRWF-TENG directly to the water separation unit, the hydrogen production rate of the whole system is 12.32 μL·min^−1^, and the sustainable hydrogen energy conversion efficiency is 2.38%.

## 5. Summary and Perspectives

In recent years, the TENG, which aims to effectively convert various environmental energies into electrical output by coupling triboelectric effect and electrostatic induction, has had its applications as a power unit and active sensors demonstrated. This review reported the recent progresses in harvesting mechanical energy from water based on the advanced TENG technology. The desirable streaming current, either on thin films or in channels, presents an excellent development and promising application prospect. The solid–solid TENG is dominated by contact electrification between two solid surfaces and operates almost exclusively by electron transfer. This mode of TENG is apt to grab ambient kinetic energy from relatively acute, even sometimes periodical, motions. In this case, the water energy harvesting is almost indirect, and the water’s kinetic energy is usually converted to the tribo-material’s motion prior to collection. With respect to the solid–liquid TENG, the formation of the EDL is the key to interfacial contact electrification. Recently, Prof. Wang proposed a new EDL model to interpret interfacial electrification that consists of the electron exchange process between liquid and solid surfaces and the interaction between ions in the liquid, which causes a gradient distribution of cations and anions near the interface. Due to the EDL-related working mechanism, solid–liquid TENGs are preferred for scavenging random and weak discrete water energy. 

Although the TENG has been demonstrated to be a promising candidate for harvesting water energy, there are still many limitations in practical application. Firstly, the TENG is characterized by the high output voltages but quite low output current and power in the current stage. This is mainly because its internal resistance is too large. More research needs to be conducted on mechanism design and material selection before TENG can be put into practical use. Secondly, corrosion resistance and waterproofing problems of TENG working in water environment energy must be solved; thus, choosing a suitable material and construction is necessary. Thirdly, how to manage and distribute output power of TENGs is also important for improving the output electrical performance of TENGs.

In the future, the continuous efforts on TENGs will largely improve their performance and real applicability in water energy harvesting through the research in the following direction.

(1) Compared with an electromagnetic generator, TENGs have an outstanding output efficiency at low frequency. TENGs are still unique for harvesting irregular and low-frequency kinetic energy, such as water waves. In order to better harvest ocean energy, more attention should be paid to the system integration and circuit management.
(2) TENG has an advantage in powering distributed microelectronics. It will be an indispensable part of the ocean sensor network and intelligent ocean research. Therefore, more efforts should be made to combine TENGs with multi-functional sensors and wireless transmission to form a self-powered ocean sensing system, which will provide a better way to monitor the marine environment.
(3) Finally, water-relevant sensor networks might be another promising route to deeply promote the advancement of TENG’s applications. In this aspect, the water-based TENGs are not only employed as water energy harvesters but also operate as active sensors for large-scale monitoring in water source, environmental variation, rainfall, and other areas.

## Figures and Tables

**Table 1:** Summary of TENGs for water wave energy harvesting.

The Mode of TENG	Structure	Year	Tribo-Layer Used in TENG	Open-Circuit Voltage (V)	Power Density and Power	References
Solid–Solid TENG	Duck-Shaped	2017	Nylon/Kapton	325	1.366 W/m^3^	[134]
	Ship-Shaped	2018	Silicone rubber/Cu	290	850 μW	[135]
	Sea Snake Structure	2018	PTFE/Nylon	55	3 W/m^3^	[132]
	Open-Book-Like TENG	2019	PTFE/Al	650	7.45 W/m^3^	[26]
	Hexagonal TENG Network	2019	FEP/Al	354	3.33 W/m^3^	[131]
	Torus Structured	2019	Nylon/FEP	83.41	0.21 W/m^3^	[136]
	Spherical	2020	FEP/Cu	250	8.5 W/m^3^	[53]
	Swing-Structured	2020	Acrylic/PTFE	342	1.29 W/m^3^	[137]
	Nodding Duck	2021	PPCF/Nylon	507	4 W/m^3^	[138]
Solid–Liquid TENG	Flexible Thin-Film TENG	2015	Water/PTFE	250	1.1 mW	[114]
	Water-FEP U-Tube TENG	2018	Water/FEP	350	2.04 W/m^3^	[139]
	Buoy-Like TENGs Network	2018	Water/PTFE	300	N.A.	[50]
	Liquid–Solid Tubular TENG	2019	Water/PTFE	N.A.	0.6 μW	[140]

**Table 2:** Summary of TENGs for water flow energy harvesting.

The Mode of TENG	Structure	Year	Tribo-Layer Used in TENG	Open-Circuit Voltage (V)	Power Density and Power	References
Solid–Solid TENG	Disk-TENG	2014	PTFE/Cu	102 (water flow of 54 mL/s)	0.03 W/m^2^	[112]
	A rotary TENG	2016	PTFE/Nylon	~102.2 (44 L/min)	~6.1 W/m^2^	[54]
	non-contact cylindrical rotating TENG	2020	FEP/Cu	1670 V (water flow of 4 L/min)	N.A.	[149]
	3D FE-TENG	2022	PTFE/Cu	150 (flow velocity of 0.81 m/s)	0.36 W/m^3^	[150]
Solid–Liquid TENG	Water-TENG	2014	Water/PTFE	72 (water flow of 54 mL/s)	0.59 W/m^2^	[112]
	scalable water wheel TENG	2019	Cu/Fluorine	N.A.	5.3 μW	[148]
